# Mineralizing Coating on 3D Printed Scaffolds for the Promotion of Osseointegration

**DOI:** 10.3389/fbioe.2022.836386

**Published:** 2022-06-27

**Authors:** Abshar Hasan, Romain Bagnol, Robert Owen, Arsalan Latif, Hassan M. Rostam, Sherif Elsharkawy, Felicity R. A. J. Rose, José Carlos Rodríguez-Cabello, Amir M. Ghaemmaghami, David Eglin, Alvaro Mata

**Affiliations:** ^1^ School of Pharmacy, University of Nottingham, Nottingham, United Kingdom; ^2^ Biodiscovery Institute, University of Nottingham, Nottingham, United Kingdom; ^3^ Department of Chemical and Environmental Engineering, University of Nottingham, Nottingham, United Kingdom; ^4^ Regenerative Orthopaedics, AO Research Institute, Davos, Switzerland; ^5^ Immunology and Immuno-Bioengineering Group, School of Life Sciences, University of Nottingham, Nottingham, United Kingdom; ^6^ Faculty of Dentistry, Oral & Craniofacial Sciences, King’s College London, London, United Kingdom; ^7^ BIOFORGE Group, University of Valladolid, CIBER-BBN, Valladolid, Spain; ^8^ Ecole des Mines Saint-Etienne, Saint-Étienne, France

**Keywords:** biomineralization, elastin-like recombinamers, bone regeneration, 3D printing, nylon, tissueimplant integration

## Abstract

Design and fabrication of implants that can perform better than autologous bone grafts remain an unmet challenge for the hard tissue regeneration in craniomaxillofacial applications. Here, we report an integrated approach combining additive manufacturing with supramolecular chemistry to develop acellular mineralizing 3D printed scaffolds for hard tissue regeneration. Our approach relies on an elastin-like recombinamer (ELR) coating designed to trigger and guide the growth of ordered apatite on the surface of 3D printed nylon scaffolds. Three test samples including a) uncoated nylon scaffolds (referred to as “Uncoated”), b) ELR coated scaffolds (referred to as “ELR only”), and c) ELR coated and *in vitro* mineralized scaffolds (referred to as “Pre-mineralized”) were prepared and tested for *in vitro* and *in vivo* performance. All test samples supported normal human immortalized mesenchymal stem cell adhesion, growth, and differentiation with enhanced cell proliferation observed in the “Pre-mineralized” samples. Using a rabbit calvarial *in vivo* model, ‘Pre-mineralized’ scaffolds also exhibited higher bone ingrowth into scaffold pores and cavities with higher tissue-implant integration. However, the coated scaffolds (“ELR only” and “Pre-mineralized”) did not exhibit significantly more new bone formation compared to “Uncoated” scaffolds. Overall, the mineralizing coating offers an opportunity to enhance integration of 3D printed bone implants. However, there is a need to further decipher and tune their immunologic response to develop truly osteoinductive/conductive surfaces.

## 1 Introduction

The demand for engineered and functional bone grafts for hard tissue repair and regeneration in craniomaxillofacial (CMF) applications is increasing due to the need for more functional designs with enhanced osseointegration (Orciani et al., 2017). Autogenous grafts are deemed to be the “gold-standard” for bone materials due to their osteoinductive, osteoconductive, and osteogenic properties (Farré-Guasch et al., 2015). However, these grafts possess several disadvantages such as donor-site morbidity, limited availability, post-operative pain, and blood loss (Aldaadaa et al., 2018). Additive manufacturing techniques offer opportunities to fabricate implants that serve as alternative grafts with advantages such as (i) complex and intricate geometrical structures, (ii) patient-specific anatomical architectures (Derby, 2012; Farré-Guasch et al., 2015), and (iii) reproducibility and cost effectiveness (Turnbull et al., 2018).

Rapid and effective osseointegration is a major goal of these types of manufactured implants. Osseointegration is an interfacial bonding phenomenon that relies on structural and functional interactions between living bone and the surface of implants during bone healing (Parithimarkalaignan and Padmanabhan, 2013). It primarily involves the growth of new bone from the native tissue towards the surface of the implant (Agarwal and García, 2015). Mechanical instability, mismatch of properties, and poor interactions at the bone-implant interface may result in non-adherent fibrous tissue formation, subsequently preventing osseointegration (Bahraminasab, 2020). In severe cases, this scenario can lead to aseptic loosening, implant failure, and adverse biological responses such as local chronic inflammation (Vallés et al., 2021). Three-dimensional (3D) printing offers the possibility of optimizing the porosity of bone implants with controlled parameters such as pore volume and diameter, pore density, and interconnectivity to promote osseointegration (Bahraminasab, 2020) by encouraging migration of bone cells and vascularization (Karageorgiou and Kaplan, 2005; Liu et al., 2020). However, 3D printed implants can suffer from a limited selection of printable materials, lack of specific chemical and physical signals to stimulate bone ingrowth and integration (Bahraminasab, 2020), poor bioactivity and control over surface roughness and texture (Tofail et al., 2018), and limited structural integrity (Ran et al., 2018).

3D printed bone constructs made from different materials to promote osseointegration have been heavily explored (Agarwal and García, 2015). CaP scaffolds have been reported to enhance osseointegration but they tend to be brittle, exhibit low compressive strengths, and display non-uniform internal structures (i.e., pore size and volume) (Wang et al., 2020). Such issues were overcome by using 3D printed metallic implants which exhibit high mechanical strength with tuneable internal structures and enhance osseointegration by increasing bone-implant interfacial strength (Petrie et al., 2009). However, they suffer from poor degradability of the implant material (Qu et al., 2019) and toxic effects caused by ions leaching from them (Prasad et al., 2017). Polymeric implants offer tunable degradability (Song et al., 2018), mechanical strength 5–10 folds better than human cancellous bone (Wang et al., 2020), and exhibit excellent biocompatibility to overcome issues related to metallic implants. However, most of the printable polymeric inks suffer from poor physio-chemical surface properties due to lack of efficient chemical functional moieties to promote cell growth and proliferation (Seyednejad et al., 2011). Thus, a variety of surface modification strategies have been investigated on polymeric scaffolds including attachment of mussel inspired polydopamine (Turnbull et al., 2018), osteogenic proteins (such as rhBMP2) (Lee et al., 2016) and mineralizing peptides (Zhang et al., 2019), and CaP coatings (Zhao et al., 2015) to enhance cell adhesion, osteogenic differentiation, and osseointegration. However, these coatings exhibit disadvantages such as propensity for proteolytic degradation in the case of peptides (Brun et al., 2013), limited bioactivity (Malhotra and Habibovic, 2016) and poor stability (Cheng et al., 2005) in the case of CaP coatings.

We have recently developed an elastin-like recombinamers (ELRs)-based mineralizing platform that can be easily coated over large and complex geometrical structures (Elsharkawy et al., 2018;Deng et al., 2021). The platform relies on the modulation of ELR order (e.g., β-sheet) and disorder (e.g., random coil) to form a supramolecular framework capable of nucleating and guiding the growth of hydroxyapatite (HAP) nanocrystals of ∼50 nm in diameter that hierarchically organize into ∼5 µm thick bundles to form mineralized macrostructures of hundreds of microns in diameter. The ELR platform can be tailored to generate different levels of apatite organization (Elsharkawy et al., 2018), to match Young’s modulus of trabecular tissue from the femoral neck (6.9 ± 4.3 GPa) to interstitial tissue from the diaphyseal cortex (25.0 ± 4.3 GPa) (Zysset et al., 1999). This capability suggests the possibility to generate mineralizing surfaces on bone implants that can be designed to match the properties of the surrounding tissue and at the same time grow apatite mineral from the implant towards the tissue, enhancing osseointegration. The mineralizing platform does not require major equipment and is simple to fabricate over large and geometrically complex structures.

In this study, we report on the integration of supramolecular chemistry, tunable organic-inorganic relationships, and additive manufacturing to engineer bone implants that can promote bone regeneration and osseointegration. We developed a simple process to uniformly coat 3D printed scaffolds while modulating ELR order-disorder ratios to trigger mineralization as a step towards osseointegration. The applicability of our coated (“ELR only” and “Pre-mineralized”) materials was assessed both *in vitro* and *in vivo* in a rabbit calvarial model. We hypothesize that our coated scaffolds can: a) attract and facilitate cell growth, b) grow mineral towards the tissue, and c) enhance integration with the surrounding tissue. We anticipate that this approach can have important implications for the design of functional dental and orthopedic implants that can self-mineralize by drawing ions from the implant site (i.e., from body fluids) to enhance bone growth and osseointegration.

## 2 Materials and Methods

### 2.1 Materials

ELR with statherin sequence (SN_A_15) were purchased from Technical Proteins Nanobiotechnology, Valladolid, Spain. Anhydrous dimethyformamide (DMF), dimethyl sulfoxide (DMSO), hexamethylene diisocyanate (HDI), calcium chloride dihydrate (CaCl_2_. 2H_2_O), sodium fluoride (NaF), and hydroxyapatite powder were procured from Sigma-Aldrich, United Kingdom. Rest of the chemicals were also procured from Sigma-Aldrich, United Kingdom unless specified.

### 2.2 3D Printed Nylon Scaffold Fabrication

**FIGURE 1:**
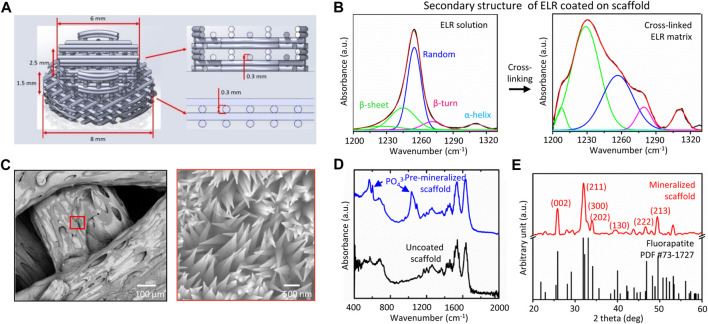
**(A)** Architecture of 3D printed nylon scaffold, **(B)** FTIR spectra showing the transition of secondary structure of the ELR from disordered (random) to ordered (β-sheet) due to solvent evaporation and crosslinking, **(C)** SEM micrographs showing mineralized structures with needle-shaped topography emerging after 14 days of scaffold mineralization, and physical characterization of the mineralized coating using **(D)** FTIR, and **(E)** XRD indicating formation of apatite mineral.

Nylon scaffolds were printed using fused deposition modeling (FDM) technique with an Ultimaker three Printer (Ultimaker, Netherlands), with a 0.4 mm diameter nozzle (Ultimaker, Netherlands) using nylon polyamide (Ultimaker, Nylon Polyamide Transparent, print temperature 240–260°C) at room temperature and ambient humidity. The printing speed was 20 mm/s for the initial layer and ranged between 10 and 12 mm/s for all other layers. The scaffold geometry was a cork like structure composed of two superposed cylinders of respectively; 8 mm diameter and 1.5 mm height with a 0°/90° alternating pattern, with a 0.3 mm layer height, and 6 mm diameter and 2.5 mm height with a 0°/0°/90°/90° pattern with a 0.3 mm layer height. The scaffold pattern was optimized to achieve lateral and vertical outer porosity of 0.3 mm and perfect fit in the bone defect (Figure 1A). First, an STL model was created using SolidWorks 2020 (Dassault Systèmes, United States). Then, the software Ultimaker Cura 4.6 (Ultimaker, Netherlands) was used to create a G-code file, which was further tested and modified until the desired dimensions and porosity, assessed with a caliper and binocular, achieved, and reproduced.

### 2.3 ELR Coating Fabrication

ELR coating on nylon scaffolds were fabricated using the procedure described previously by our group (Elsharkawy et al., 2018). Briefly, lyophilized ELR powder was dissolved in solvent mixture of DMF/DMSO (at 9/1 ratio) to prepare 5% (w/v) ELR solution followed by addition of hexamethyl diisocyanate (HDI) crosslinker (cross-linker to lysine ratios of 12/1). Finally, 3D printed nylon scaffolds were dipped in the above ELR solution for 10–15 s and later left for drying overnight at room temperature (22°C) inside a glovebox (BELLE Technology, United Kingdom) maintained at a humidity <20%. Dried and ELR coated scaffolds washed several times with de-ionized water to remove excess HDI and stored at 4°C until use and were termed as “ELR only” scaffold.

### 2.4 Mineralization Experiment

Mineralizing solution was prepared using previously reported methodology (Elsharkawy et al., 2018). Briefly, hydroxyapatite powder (2 mM) and sodium fluoride (2 mM) were dissolved in de-ionized water by dropwise adding nitric acid (69%, v/v) into the solution until it becomes clear. The pH of the above solution was adjusted to 6.0 using 30% (v/v) ammonium hydroxide solution. To create ‘Pre-mineralized’ scaffolds, “ELR only” scaffolds were incubated in above solution (pH = 6) at 37°C for 2 weeks. Post mineralization, scaffolds were washed several times with deionized water (diH_2_O), air dried, and stored at 37°C until use.

### 2.5 Characterization

#### 2.5.1 Scanning Electron Microscopy

“Pre-mineralized” scaffold sample were mounted on aluminum stubs using double sided carbon tape followed by 10 nm thick Iridium coating (Model: 150T ES, Quorum, United Kingdom) to make the sample conductive. Surface topographies of mineralized scaffold samples were analyzed using JEOL 7100F scanning electron microscopy (JEOL, United Kingdom) operated at 15 kV. Scaffolds were handled gently using Teflon tweezers to prevent any damage to the coating.

#### 2.5.2 Attenuated Total Reflection-Fourier-Transform Infrared Spectroscopy

ATR-FTIR spectroscopy analysis of “ELR only” and “Pre-mineralized” scaffolds before and after *in vitro* mineralization was carried out using Cary 630 FTIR Spectrometer (Agilent, United Kingdom). Sixty four scans on average were recorded for each sample type at a resolution of 2 cm^−1^ in the range 4000–450 cm^−1^. The obtained spectra were analyzed by Origin 8.5 software to make the spectrum curve.

#### 2.5.3 X-Ray Diffraction

XRD scans were recorded for phase Identification and quantification of the “Pre-mineralized” scaffold using D8 Advance with DaVinci X-ray diffractometer (Bruker, United Kingdom). Instrument was operated with flat plate θ/θ geometry and Ni-filtered Cu-Kα radiation at 45 kV and 40 mA (Kα1 = 1.54059 Å, Kα1 = 1.54442 Å) (Elsharkawy et al., 2018). The values were recorded from 5° to 70° with a step size 0.02°, and data were obtained at step time of 1,600 s. PDF4 database (ICDD, USA, release 2014) was used for comparison.

### 2.6 *In Vitro* Studies

Human immortalized mesenchymal stem cells (hiMSCs) were generated in-house by lentiviral transfection of E6/E7 and hTERT genes as previously described (Mori et al., 2005; Balducci et al., 2014; Burroughs et al., 2021). Cells were cultured in basal media (BM) composed from Dulbecco’s modified Eagle’s medium (DMEM) supplemented with 10% (v/v) fetal bovine serum (FBS) and 1% (v/v) penicillin-streptomycin. Test samples were sterilized by submerging in 70% ethanol for 30 min then washing three times in sterile 1X phosphate buffer saline (PBS). They were then transferred to individual wells of a 96-well plate and exposed to UV for an hour to ensure complete sterilization. Test samples were then soaked in BM for 1 h to permit protein adsorption and promote cell attachment. To seed, hiMSCs were added at a density of 10,000 per cm^2^ (2,800 per disc) at a concentration of 14,000 cells/mL (200 µL per disc) in BM. After 48 h, discs were transferred to a new 96-well plate to conserve only adhered cells. Later, 200 µL osteogenic induction media (OIM) consisting of BM supplemented with 100 nM dexamethanone, 50 μg/ml ascorbic acid 2-phosphate and 5 mM β-glycerophosphate was added to each well and considered as day 1. Media was changed every 2–3 days and cells were maintained in a humidified incubator at 37°C and 5% CO_2_ in air. Quadruplicate of each sample type was used for each of the *in vitro* experiment described below.

#### 2.6.1 Metabolic Activity

To assess viability, metabolic activity was measured on days 1, 8 and 15 using PrestoBlue® (ThermoFisher Scientific, United Kingdom). Briefly, culture media was replaced with 200 µL of PrestoBlue® working solution (10% PrestoBlue® in BM) and incubated for 1 h. The solution was then transferred to a black 96-well plate and read at λ_ex_: 560 nm, λ_em_: 590 nm in a plate reader (Tecan Infinite 200, Switzerland), where fluorescence correlates with metabolic activity, and fresh OIM was added to the discs. Each group consisted of five samples (n = 5).

#### 2.6.2 Alkaline Phosphatase Activity and Total DNA Quantification

To assess osteogenic differentiation, ALP activity and total DNA was quantified on days 8 and 15 using cell lysates as previously described (Owen et al., 2020). Briefly, to digest, media was removed, and the discs were washed with PBS before transferring to a microcentrifuge tube containing 500 µL of cell digestion buffer (10 vol% cell assay buffer (1.5 M Tris-HCl, 1 Mm ZnCl_2_, 1 mM MgCl_2_ in diH_2_O, 1% Triton-X100 in diH_2_O)). Samples (*n* = 5) were refrigerated for 1 h before freeze-thawing three times (−80°C/37°C, centrifuging (10,000 RCF) for 5 min and homogenizing the supernatant. ALP activity was determined using the Pierce^TM^ PNPP substrate kit (ThermoFisher Scientific, United Kingdom) according to the manufacturer’s instructions. Briefly, 20 μL of lysate was combined with 180 μL of substrate (p-nitrophenol phosphate, pNPP) in a 96-well plate. The change in absorbance was measured using a plate reader (Tecan infinite 200) at a wavelength of 405 nm every minute for 30 min. The ALP activity is expressed as nmol of p-nitrophenol per minute (nmol pNP/min), assuming that one absorbance value equals 25.2 nmol of product. This activity was normalized to the total DNA content per lysate. DNA was quantified using the Quant-it^TM^ high sensitivity dsDNA kit (ThermoFisher Scientific, United Kingdom), according to manufacturer’s instructions. Briefly, 20 μL of lysate was combined with 180 μL of substrate in a black 96-well plate. The plates were shaken to aid the DNA-substrate conjugation, left at room temperature for 10 min, then shaken again before measuring the fluorescence (λex:485nm,λem:535nm) . The shaking and fluorescence were performed and measured using a plate reader (Tecan infinite 200, Switzerland). The fluorescence was converted to ng of DNA using a standard curve and was scaled to the total lysate volume. Each group consisted of five samples (*n* = 5).

#### 2.6.3 Fluorescence Imaging

Cell growth on the discs (sample size for each group, *n* = 5) was visualized on day 5 using fluorescence microscopy. Day 5 was chosen over day 8 as the adhered cells were too confluent after 8 days of culture to distinguish the effect of substrates on cell spreading and morphology. To fix, media was removed, and discs were washed twice with PBS before submerging in 3.7% formaldehyde for 20 min. To stain, fixed discs were washed twice in PBS then submerged in immunocytochemistry (ICC) buffer (1% BSA, 0.1% Triton-X100 in PBS) containing 1X Phalloidin-iFluor™ 633 (Stratech, United Kingdom) for 1 h at room temperature. Discs were then washed in PBS before imaging. Images of hiMSCs on the disc surfaces (2048 × 2048 pixels) were obtained using a Leica TCS LSI (Leica Microsystems, United Kingdom) at λ_ex_: 635 nm, λ_em_: 650 nm.

### 2.7 *In Vivo* Studies

The osteogenesis inducing capacity of scaffolds was analyzed *in vivo* using 6 mm critical-size calvarial defect model in six female New Zealand white rabbits at AO research institute Davos, Switzerland. A total of four calvarial defects were created per animal and each animal had all three types of test groups (i.e., “Uncoated,” “ELR only,” and “Pre-mineralized” scaffolds) and positive control (Bio-Oss). Thus, sample size for each test groups and positive control groups was 6. The negative control group (empty defect) was retrieved from previous studies performed at the AO research institute. Scaffolds were handled gently using Teflon tweezers to prevent any damage to the coating. Scaffolds were ethylene oxide sterilized prior to implantation. The animals were housed singly and received food and water *ad libitum*. All animals’ research protocols were approved (Approval ref. No. 21) by the Animal Welfare & Ethical Review Body (AWERB) at the University of Nottingham and at the AO Research Institute Davos.

#### 2.7.1 Surgical Intervention

The rabbits were sedated with a combination of medetomidine, midazolam, and fentanyl in the preparation area approximately 20 min before starting the aseptic preparation of the surgical field. A skin incision was made on midline of the caudal dorsal skull from the nasal bone to the occipital crest using a #10 scalpel blade. A bone cutting jig was placed on midline of the parietal bone, spanning the left and right parietal bones just caudal to the horizontal suture line. The locations of four evenly distributed defects were marked using blunt dissection of the periosteum through the jig using a #15 scalpel blade. Four 6 mm diameter cranial defects were created in the parietal bone with an Anspach® drill associated with a Codman perforator (DePuy Synthes, United States) using procedure described previously (Guillaume et al., 2019). Any remaining bone pieces were gently removed from the defects without damaging the dura mater. The hydrated scaffolds were fitted into the calvarial defects according to their respective study groups. A total of four calvarial defects were created per animal and each animal had all three types of test groups (i.e., “Uncoated,” “ELR only,” and “Pre-mineralized” scaffolds) and a positive control (Bio-Oss). The subcutaneous tissues were closed with 4–0 Monocryl in a simple interrupted pattern, and the skin is closed using 5-0 vicryl rapide in an intradermal pattern. The animals were postoperatively scanned in the Xtreme CT. Fluorochromes (Calcein green (1 ml/kg) and xylenol orange (1 ml/kg)) were administered at 2 and 4 weeks postoperatively for evaluation of new bone formation using histological analysis after euthanasia. The animals were euthanized after 6 weeks postoperatively by means of an intravenous overdose of barbiturate (Pentobarbital, Esconarkon®).

#### 2.7.2 High-Resolution Micro-Computed Tomography Analysis

Micro-CT scans were recorded immediately after euthanasia *in situ* using high-resolution peripheral quantitative computed tomography (HR-pQCT) (Model: XTremeCT-II, Scanco Medical AG, Switzerland). The parameters of the scan were: voltage source 81 kV, current source 124 mA, image pixel size 9 mm, an aluminum filter of 0.5 mm, a tomographic rotation of 180°, and a sample rotation step of 0.8°. Later, the samples were fixed in 4% buffered formalin and examined under vivaCT (voltage source: 60 kV, current source: 900 μA, image pixel size: 82 μm, a tomographic rotation of 180°, and a sample rotation step of 0.4°) for the individual calvarial defects. A cylindrical volume of interest (VOI) was used to quantify the bone volume and bone mineral density corresponding with the size of the defect.

#### 2.7.3 Histology Analysis

Histology analysis was performed using procedure reported previously by our group (Tejeda-Montes et al., 2014a). Briefly, the skull calvarias were extracted and fixed in 4% buffered formalin at pH = 7.2 for 2 days followed by bone decalcification suing Surgipath Decalcifier II for 4 h. Later, they were embedded in paraffin and sectioned using microtome to prepare 3 mm thick sections and stained with hematoxylin and eosin (H&E) to observe under a microscope Zeiss AxioScope A (Carl Zeiss) with a Zeiss AxioCam MRc 5 camera (Carl Zeiss, Madrid, Spain) for qualitative and semiquantitative evaluation.

### 2.8 *In Vitro* Immunological Analysis

Monocytes were isolated and cultured using procedure developed previously (Awuah et al., 2019). Briefly, buffy coats were procured from healthy donors following approval (REC 260 - 1701) from ethics committee (Research Ethics Committee, Faculty of Medicine and Health Sciences, University of Nottingham). Monocytes were isolated from peripheral blood mononuclear cells. A MACS magnetic cell separation system (CD14 MicroBeads positive selection with LS columns, Miltenyi Biotec) was used for the isolation as previously described (Salazar et al., 2016). The obtained monocytes using this method exhibited ∼95% purity as analyzed by CD14 expression. Monocytes (1 × 10^6^ cells/mL) were prepared and cultured in RPMI-1640 medium at 37°C, 5% CO_2_ in a humidified incubator and 250 µL of the cell suspensions were seeded on pre-sterilized test samples (sample size, *n* = 6) for 3 and 6 days. Post incubation, the level of IL-10 secreted into the media by macrophages was quantified by sandwich ELISA using DuoSet ELISA development kits (R&D Systems, United States) as per manufacturer’s instructions.

### 2.9 Statistical Analysis

All the data are reported as mean ± SD. Statistical analysis was performed using GraphPad Prism ver. 6 software between the means of different test groups using one-way and two-way analysis of variance (ANOVA) with the Tukey test. *p* values <0.05 were considered significant.

## 3 Results and Discussions

### 3.1 Rationale of Design

Implant-tissue integration or osseointegration is critical for the success and function of implants. Osseointegration is defined as a formation of a direct interface between an orthopedic or dental implant and bone, without intervening soft tissue (Albrektsson and Albrektsson, 1987). 3D printed polymeric scaffolds can promote cell growth, differentiation, and biomineral formation, however, exhibit poor integration with the surrounding tissue (Jackson et al., 2018). Here, our study aims to integrate an ELR based self-mineralizing coating (by drawing Ca and P ions from the implant site) with 3D printed nylon scaffold for applications in bone repair and regeneration. Thus, the objectives of the study are: (a) fabrication and optimization of the 3D printed nylon scaffolds with high porosity, (b) optimization of ELR coating on the scaffolds, (c) assessment of the applicability of our coated (“ELR only” and “Pre-mineralized”) materials both *in vitro* and *in vivo* in a rabbit calvarial model. We hypothesize that these scaffolds: a) can attract and facilitate cell growth, b) can grow mineral towards the tissue, and c) can enhance integration with the surrounding tissue. We developed a simple process to uniformly coat 3D printed scaffolds while modulating ELR order-disorder ratios to trigger mineralization as a step towards osseointegration. To investigate the role of the growing mineral on the surface of the scaffolds, experiments were conducted using ELR-coated scaffolds that were either fully mineralized (“Pre-mineralized”) or non-mineralized (“ELR only”). The ELRs comprised hydrophobic (VPGIG) and hydrophilic (VPGKG) moieties that enable modulation of secondary structure and optimization of order-disorder rations to trigger mineralization as we previously demonstrated (Elsharkawy et al., 2018). Cells (hiMSCs) were used to assess the capacity of the mineralized surfaces to promote adhesion, proliferation, and differentiation *in vitro* while a rabbit calvarial model was used to assess bone regeneration and bone-implant integration *in vivo*. We also performed preliminary *in vitro* experiments using monocyte-derived macrophages to provide insights into the potential immunomodulatory effects of the mineralized coatings.

### 3.2 Coating Characterization and Mineralization

The pore size and porosity of implants are known to significantly influence bone formation and integration with the surrounding tissue. Thus, we designed our nylon scaffolds with pore diameters ranging between 300–600 μm, which is reported to be optimum for bone ingrowth (Mehrabanian and Nasr-Esfahani, 2011). 3D printed nylon scaffolds were coated with 5–10 µm thick ELR coating and were characterized for secondary structure composition using FTIR. In solution, the ELR exhibits a secondary structure consisting of a random (disordered) to β-sheet (ordered) ratio of 6.84 ± 0.71 (Figure 1B). Upon solvent evaporation, the resulting coating exhibit a secondary structure consisting of disordered to ordered ratio of 0.47 ± 0.04. These values are aligned with those reported previously by our group on mineralizing membranes (Elsharkawy et al., 2018). ELR coated scaffolds were mineralized *in vitro* for 2 weeks and characterized for mineral growth. SEM micrographs of the mineralized scaffolds depicted well defined crystals with needle shape morphology nucleating and growing on the surface of the scaffolds (Figure 1C). Mineralization was confirmed by FTIR spectroscopy (Figure 1D) and XRD (Figure 1E) analysis displaying non-stoichiometric apatite spectral peaks that demonstrate a crystalline phase and structural parameters similar to fluorapatite, respectively, as previously reported (Elsharkawy et al., 2018; Deng et al., 2021).

### 3.3 *In Vitro* Studies

All test samples were first evaluated *via in vitro* cell-based assays and using hiMSCs.

#### 3.3.1 Metabolic Activity Analysis

**FIGURE 2:**
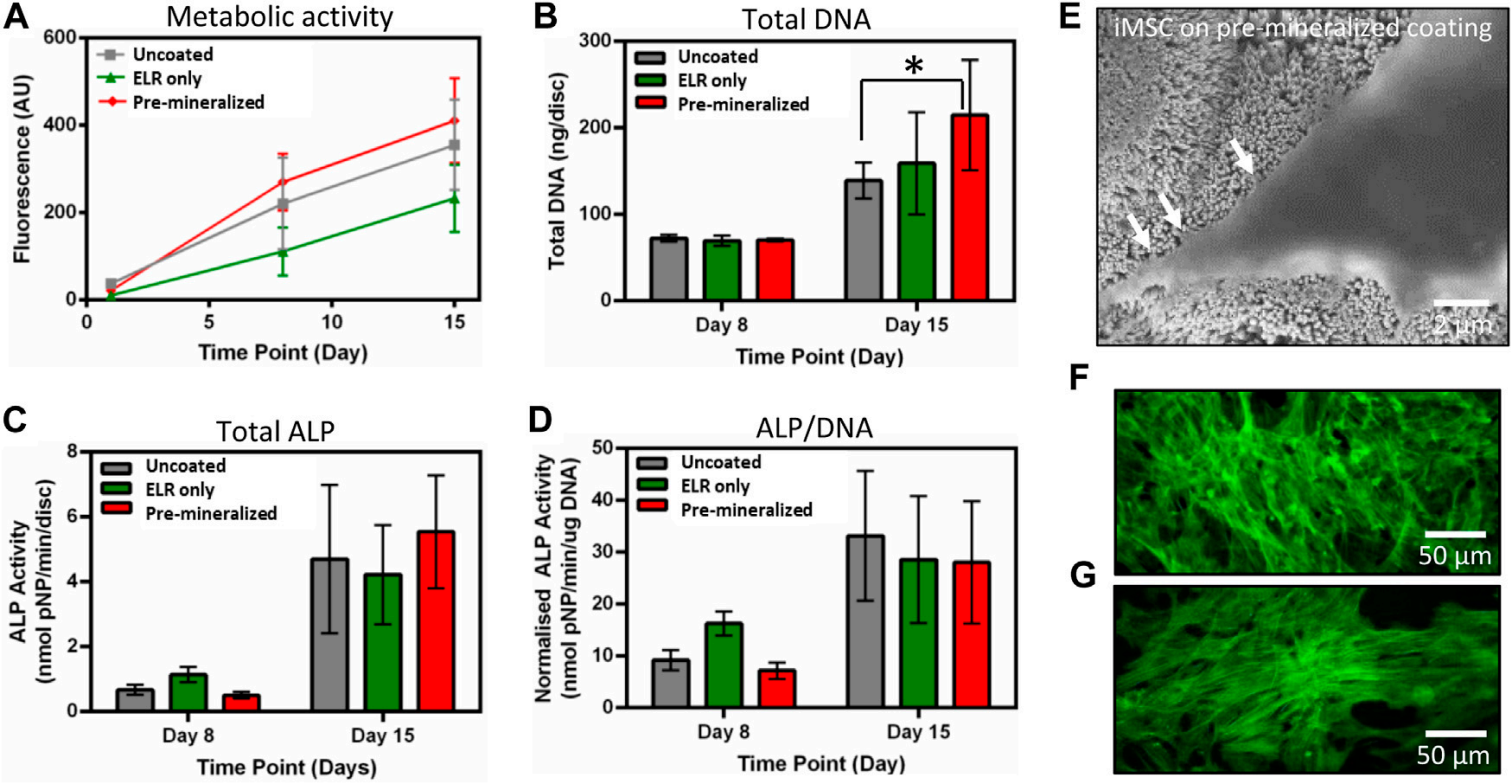
*In vitro* characterization. **(A)** Metabolic activity, **(B)** total DNA, **(C)** total ALP, and **(D)** normalized ALP activity of hiMSCs on different test samples. **(E)** SEM micrographs of hiMSCs after 5 days of culture on “Pre-mineralized” samples depicting cell protrusions (as pointed by arrow heads) that indicate cell spreading and migration, and fluorescence microscopic images of hiMSCs cultured for 5 days on **(F)** “Pre-mineralized,” and **(G)** “ELR only” coated samples. Data presented at mean ± SD (*n* = 6). In **(B)** * represents significant difference *p* < 0.05 between “Uncoated” and “Pre-mineralized” scaffold, estimated using one-way ANOVA in GraphPad Prism ver. 6 software.

We performed metabolic activity analysis on different test samples using non-toxic PrestoBlue® at 1, 8, and 15 days after cell seeding. We observed that metabolic activity increased at a similar rate on all test samples, as indicated by the similar gradients. However, metabolic activity was significantly lower (*p* < 0.05) on “ELR only” at days 8 and 15 in comparison to “Uncoated” and “Pre-mineralized” (Figure 2A). We speculate that the observed lower metabolic activity on “ELR only” coated surfaces may result from the more hydrophobic nature of “ELR only” samples. Surface hydrophilicity plays a crucial role in controlling protein adsorption and conformation (Hasan et al., 2018) that in turns regulate cell adhesion and proliferation (Hasan et al., 2018; Hasan and Pandey, 2020). Hydrophobic surfaces are known to exhibit irreversible adsorption of ECM proteins (such as fibronectin, vitronectin, collagen) that leads to protein denaturation and consequently negative effects on cell adhesion (Cai et al., 2020). As the “ELR only” coating is markedly very hydrophobic (water contact angle = 115°) (Tejeda-Montes et al., 2012) than the ‘Pre-mineralized’ coating (water contact angle = 41° ± 9°), it is possible that this effect could lead to cells having lower metabolic activity and cell proliferation on “ELR only” coatings.

#### 3.3.2 Cell Adhesion and Proliferation

Total DNA was quantified on days 8 and 15 as a measure of the number of hiMSCs on the samples and proliferation between the timepoints. Cells were harvested and DNA extracted then quantified using the Quant-iTTM high sensitivity dsDNA kit. While there was no difference between all samples on day 8, ‘Pre-mineralized’ samples exhibited significantly higher total DNA quantity (*p* < 0.05) by day 15 (Figure 2B). Higher values of DNA extracted from “Pre-mineralized” surfaces indicate enhanced cell proliferation as compared to the other samples. We attribute this enhanced level of total DNA to the bioactive nature of CaP mineral (Jeong et al., 2019) which has been reported to promote osseointegration (Zhu et al., 2021).

#### 3.3.3 Alkaline Phosphatase Assay

ALP is an early ostegenic marker and is an enzyme associated with osteogenesis. It is expressed by MSCs as they undergo osteogeneic differentiation and plays an essential role in matrix mineralization (Burroughs et al., 2021). Therefore, here, early osteoblast differentiation was characterized using an ALP assay normalized to DNA content. ALP activity increased after 8 and 15 days on all test samples. However, there was no statistical difference observed in total (Figure 2C) or normalized ALP (Figure 2D) between the samples, which indicates that cell exhibited similar differentiation response irrespective of the substrate type and suggests no negative effect on osteogenesis.

#### 3.3.4 Cell Morphology

SEM and fluorescent imaging of adhered cells at day 5 revealed cell morphology with elongated shapes indicating good cellular attachment and spreading across all samples (Figures 2E,F,G). These results are consistent with the higher proliferation results (Figure 2B). Higher cell spreading with cellular extensions *in vitro* indicate cell migration which is crucial for bone tissue healing and regeneration (Fu et al., 2019).

Overall, these *in vitro* results indicate that all test samples are able to support normal hiMSCs performance with no negative effects observed on cell adhesion, growth, and differentiation. However, it is important to point the enhanced proliferation observed in the mineralized samples, suggesting the potential of the coating to promote cell *growth in vivo.*

### 3.4 *In Vivo* Studies

**FIGURE 3:**
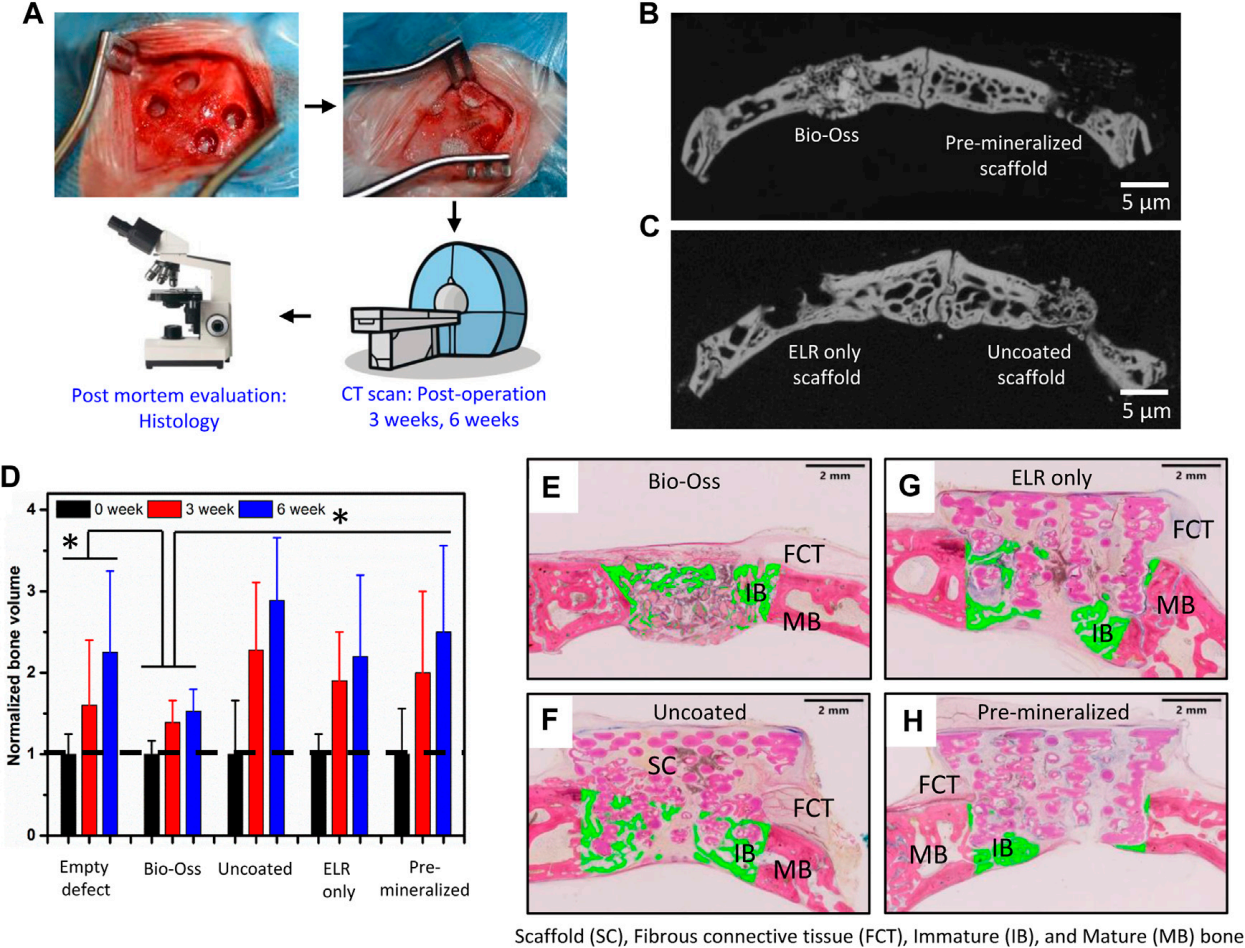
*In vivo* characterization. **(A)** Schematic of the study plan and view of the rabbit calvarial bone defect before and after implantation. Micro CT images of new bone formation in [**(B)**, left] the positive control (Bio-Oss) and [**(B)**, right] “Pre-mineralized” scaffold and [**(C)**, left] “ELR only” and [**(C)**, right] “Uncoated” scaffolds. **(D)** Normalized volume of newly formed bone with different test samples after 0, 3, and 6 weeks of implantation. Histological sections stained with Giemsa-Eosin depicting new bone formation marked with green colour after 6 weeks of implantation including **(E)** positive control (Bio-Oss), **(F)** “Uncoated” nylon scaffold, **(G)** “ELR only” coated nylon scaffold, and **(H)** “Pre-mineralized” scaffold. Scaffold (SC), Fibrous connective tissue (FCT), Immature (IB) and Mature (MB) bone. In **(D)** * represents significant difference *p* < 0.05 in normalized bone volume between sample groups and at different time points, estimated using two-way ANOVA in GraphPad Prism ver. 6 software.

Given the observed *in vitro* mineralizing capacity and osteogenic differentiation of hiMSCs cells, the bone regeneration and infiltration capacity of the different test groups was investigated *in vivo* using an orthotopic 6 mm wide calvarial bone defect model in rabbits (Figure 3A). Calvarial bone defect model involves formation of bilateral round shaped defects in the parietal bone which can vary in size from 6–10 mm in diameter (Lee et al., 2010; Schmidlin et al., 2013; Bisht et al., 2021). Bone ossification was assessed by micro-CT and histology using Giemsa-Eosin staining after 6 weeks of implantation. The micro-CT analysis demonstrated that all tested samples exhibited new bone formation after 3 and 6 weeks of implantation. However, no significant difference in new bone volume within the defect among the test groups “Uncoated,” “ELR only,” and “Pre-mineralized” (Figures 3B,C) was quantified using micro-CT nor qualitatively observed *via* histology. The positive control Bio-Oss exhibited the lowest ossified tissue within the defect (Figure 3D). We speculate that this may result from a dense calcified material in large amount in the defect, which do not significantly degrade within the 6 week period of the experiment (Bosetti et al., 2013) and may consequently require less time to reach full bone defect healing.

**FIGURE 4:**
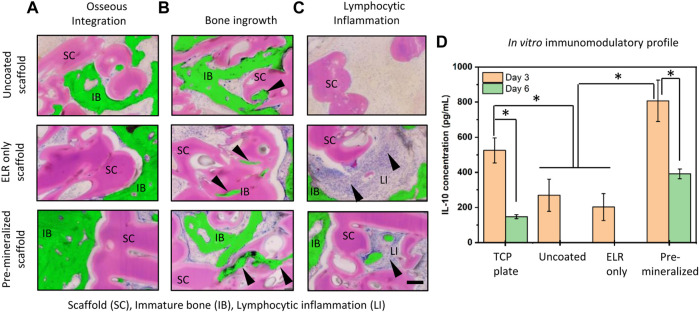
Histological sections stained with Giemsa-Eosin depicting **(A)** osseous interaction with the implant at the bone-implant interface, **(B)** bone ingrowth into small pores and cavities of the scaffolds, and **(C)** lymphocytic inflammation with infiltrating cells (arrow heads) primarily around “ELR only” coating and “Pre-mineralized” scaffolds. Newly formed bone in the histology images have been pseudo colored and represented with green to show clear difference between immature (new) bone and scaffold. Scale bar = 200 µm. Scaffold (SC), Immature bone (IB), Lymphocytic inflammation (LI). **(D)** Estimation of IL-10 concentrations secreted from macrophages after 3 and 6 days of culture on different test samples. * represents significant difference *p* < 0.005, estimated using two-way ANOVA in GraphPad Prism ver. 6 software.

From the Giemsa-Eosin-stained histological sections (Figures 3E–H), all test groups exhibited bone regeneration along the rim region of the defects, with higher levels of ossified tissue at the center of defects treated with “Uncoated” and “ELR only” (Figures 3F,G). Nylon-based scaffolds have been shown to support pre-osteoblasts cells adhesion and proliferation (Abdal-hay et al., 2015) and we have previously showed that the ELR material, which contains the statherin-derived amino acid sequence DDDEEKFLRRIGRFG (SN_A_15) known to promote HAP formation in the oral environment (Hay and Moreno, 2021), can stimulate osteoblastic differentiation *in vitro* (Tejeda-Montes et al., 2014b) and bone formation *in vivo* (Tejeda-Montes et al., 2014a). Furthermore, histology results revealed higher conformation of the new bone tissue to the scaffold’s geometry in “Pre-mineralized” scaffolds (Figure 4A) as compared to the other test groups. This was evident by the presence of undulations which indicate newly formed bone conforming tightly to and taking the shape of the architecture of the scaffold. This behavior of formation of bony undulations at the implant surface in response to the surface physio-chemical properties and implant’s geometry, referred as contact osteogenesis (Shah et al., 2019) indicates firm anchorage of the newly formed bone to the implant surface (Khosravi et al., 2018; Shah et al., 2019). When investigating bone ingrowth into small pores and cavities within the scaffolds, “Uncoated” and “Pre-mineralized” scaffolds exhibited more bone in-growth as compared to “ELR only” (Figure 4B). Moreover, we did not observe any signs of fibrous tissue formation at the implant-tissue interface on all our scaffold types (i.e., “Uncoated,” “ELR only,” and “Pre-mineralized”) (Figures 4A,B). This is one of the characteristic of osseointegrated implants (Shah et al., 2019). Fibrous tissue formation is a surface responsive behavior. For instance, stiff surfaces can activate myofibroblasts (a scar-forming cell type) that leads to fibrous formation around the implant (Noskovicova et al., 2021b), thus, blocking implant-tissue integration (Noskovicova et al., 2021a). It is possible that a similar effect takes place at the surface of all our scaffold types (i.e., “Uncoated,” “ELR only,” and “Pre-mineralized”) avoiding activation of myofibroblasts and thus preventing fibrous tissue formation. However, more in-depth characterization such as (i) biomechanical analysis of implant-tissue interlocking (Brånemark et al., 1998) and (ii) high resolution electron tomography at implant-tissue interface to understand bone structure arrangement at nanoscale (Wang et al., 2017) would be required in further studies to gain more insights into osseointegration.

### 3.5 *In Vitro* Immunomodulatory Profile

Interestingly, we observed signs of inflammation with infiltrating lymphocytic cells (arrow heads) near the implant site of “ELR only” and more pronounced in “Pre-mineralized” scaffolds (Figure 4C). Presence of these inflammatory lymphocytic cells at the implant site (Figure 4C) indicates positive response to bone healing and osseointegration (Andrew et al., 1994; Trindade et al., 2016; Davies, 2019). Furthermore, lymphocyte cells are known to play crucial role in collagen deposition and organization during bone matrix formation in fracture healing (El Khassawna et al., 2017). HAP particles especially with needle-shape morphology (Lebre et al., 2017) and HAP coatings (Jiang et al., 2022) are known to exhibit excellent *in vivo* osteoimmunomodulatory properties. Inspired by these reported observations, we anticipated that the needle-shaped topographies generated by the mineralized material on the surface of our “Pre-mineralized” scaffolds may be playing an immunomodulatory role and thus motivated us to gain more insight into this potential effect. Therefore, we cultured human monocyte derived macrophages on different scaffolds and quantified IL-10 secretion using sandwich ELISA (Figure 4D). IL-10 is a potent anti-inflammatory cytokine secreted by lymphocytes, macrophages, and dendritic cells, which is known to suppresses both immunoproliferative and inflammatory responses and plays a critical role in bone healing and remodeling (Jung et al., 2013) by inhibiting osteoclastic bone resorption and promoting osteoblastic bone formation (Zhang et al., 2014). Our results demonstrate that “Pre-mineralized” coatings exhibited significantly higher levels (807 ± 117 pg/ml) of IL-10 (*p* < 0.005) on day 3 which later dropped to lower levels (391 ± 28 pg/ml) after 6 days. Interestingly, we observed significantly lower concentrations of IL-10 on “Uncoated” (270 ± 92 pg/ml) and “ELR only” (202 ± 76 pg/ml) coatings as compared to “Pre-mineralized” coating at day 3 of culturing (*p* < 0.005) and IL-10 was undetectable at day 6 (Figure 4D). Overall, all test samples exhibited IL-10 concentrations which lie in the range which promotes bone healing, as reported previously (Chen et al., 2018). Previous studies in mice have shown that IL-10 deficiency can lead to poor bone formation and osteoblastogenesis, resulting in osteopenia and high bone fragility (Dresner-Pollak et al., 2004; Holgersen et al., 2015). However, it is crucial to note that the effect of IL-10 on osteogenesis is concentration dependent. For instance, low concentrations of IL-10 (10–1,000 pg/ml) promote osteogenesis *via* p38/MAPK signaling pathway, whereas higher concentrations (10,000–100,000 pg/ml) activate NF-kB to downregulate p38/MAPK signaling, thus inhibiting osteogenesis (Chen et al., 2018). These results demonstrate that the mineralized coating is having a significant effect on IL-10 production and is likely leading to a different immunomodulatory response *in vivo* compared to the other groups tested. While a more in-depth analysis of this effect is important to understand these immunomodulatory effects, this work is beyond the scope of the current study.

## 4 Conclusion

The present work reports on the possibility of integrating supramolecular chemistry and additive manufacturing to engineer and fabricate functional bone implants that can promote bone regeneration. 3D printed nylon scaffolds were coated with mineralizing ELR matrix and were assessed both *in vitro* and *in vivo* using a rabbit calvarial model for bone formation and osseointegration. Our results indicate that the mineral grown was apatite in nature and grew uniformly over large and uneven area of the scaffold. *In vitro*, all test samples (“Uncoated,” “ELR only,” and “Pre-mineralized”) supported hiMSCs adhesion, proliferation, and spreading of hiMSCs cells growing preferentially on “Pre-mineralized” samples. *In vivo*, all test samples exhibited higher levels of new bone formed within the defect compared to the control Bio-Oss. However, coated scaffolds (both “ELR only” and “Pre-mineralized”) did not lead to higher bone formation compared to “Uncoated” scaffolds.

In conclusion, our mineralizing coatings offer higher cell response *in vitro*, qualitatively higher conformation of the new bone tissue to the geometry of the scaffold, and no fibrous tissue formation at the implant-tissue interface. However, this study exhibit limitations that could be improved. For example, the coatings need to be optimized as they did not significantly enhance the volume of the newly formed bone. Furthermore, optimization of immunomodulation and in-depth integration analysis between tissue and scaffold need to be performed. Therefore, future studies should be aimed at (i) optimizing the coatings (ii) optimizing the architecture of the scaffold, (iii) modulating the morphology of the HAP structures, (iv) assessing *in vivo* performance for longer periods of time to investigate mineral growth from the scaffold to the tissue, (v) characterizing implant-tissue inter-locking, and (vi) optimizing immunomodulation. It is important to mention that the supramolecular organization of the ELR molecules can be tailored during the coating process to modify and optimize the growth of the inorganic phase (Elsharkawy et al., 2018). In addition to this optimization to attempt to enhance osseointegration, degradability and absorbability of the material should also be characterized in future studies.

Overall, our results indicate the potential of the coatings to promote responses that can ultimately led to osseointegration. We envisage that this approach can have important implications for the design of smart biomaterials which can acellularly self-mineralize by drawing ions from the implant site and exhibit the capacity to enhance bone growth and osseointegration.

## Data Availability

The original contributions presented in the study are included in the article/Supplementary Material, further inquiries can be directed to the corresponding authors.

## References

[B1] Abdal-hay A. Hamdy A. S. Khalil K. A. (2015). Fabrication of Durable High Performance Hybrid Nanofiber Scaffolds for Bone Tissue Regeneration Using a Novel, Simple *In Situ* Deposition Approach of Polyvinyl Alcohol on Electrospun Nylon 6 Nanofibers. Mater. Lett. 147, 25–28. 10.1016/j.matlet.2015.02.005

[B2] Agarwal R. García A. J. (2015). Biomaterial Strategies for Engineering Implants for Enhanced Osseointegration and Bone Repair. Adv. drug Deliv. Rev. 94, 53–62. 10.1016/j.addr.2015.03.013 25861724 PMC4598264

[B3] Albrektsson T. Albrektsson B. (1987). Osseointegration of Bone Implants: A Review of an Alternative Mode of Fixation. Acta Orthop. Scand. 58, 567–577. 10.3109/17453678709146401 3321881

[B4] Aldaadaa A. Owji N. Knowles J. (2018). Three-dimensional Printing in Maxillofacial Surgery: Hype versus Reality. J. Tissue Eng. 9, 2041731418770909. 10.1177/2041731418770909 29774140 PMC5949934

[B5] Andrew J. G. Andrew S. Freemont A. Marsh D. (1994). Inflammatory Cells in Normal Human Fracture Healing. Sort 65, 462–466. 10.3109/17453679408995493 7976298

[B6] Awuah D. Alobaid M. Latif A. Salazar F. Emes R. D. Ghaemmaghami A. M. (2019). The Cross-Talk between miR-511-3p and C-type Lectin Receptors on Dendritic Cells Affects Dendritic Cell Function. J. I. 203, 148–157. 10.4049/jimmunol.1801108 31118225

[B7] Bahraminasab M. (2020). Challenges on Optimization of 3D-Printed Bone Scaffolds. Biomed. Eng. Online 19, 69. 10.1186/s12938-020-00810-2 32883300 PMC7469110

[B8] Balducci L. Blasi A. Saldarelli M. Soleti A. Pessina A. Bonomi A. (2014). Immortalization of Human Adipose-Derived Stromal Cells: Production of Cell Lines with High Growth Rate, Mesenchymal Marker Expression and Capability to Secrete High Levels of Angiogenic Factors. Stem Cell Res. Ther. 5, 63. 10.1186/scrt452 24887516 PMC4055112

[B9] Bisht B. Hope A. Mukherjee A. Paul M. K. (2021). Advances in the Fabrication of Scaffold and 3D Printing of Biomimetic Bone Graft. Ann. Biomed. Eng. 49 (4), 1128–1150. 10.1007/s10439-021-02752-9 33674908

[B10] Bosetti M. Bianchi A. E. Zaffe D. Cannas M. (2013). Comparative *In Vitro* Study of Four Commercial Biomaterials Used for Bone Grafting. J. Appl. biomaterials Funct. Mater. 11, 80–88. 10.5301/jabfm.5000149 23728538

[B11] Brånemark R. Öhrnell L. O. Skalak R. Carlsson L. Brånemark P. I. (1998). Biomechanical Characterization of Osseointegration: An Experimental *In Vivo* Investigation in the Beagle Dog. J. Orthop. Res. 16, 61–69. 9565075 10.1002/jor.1100160111

[B12] Brun P. Scorzeto M. Vassanelli S. Castagliuolo I. Palù G. Ghezzo F. (2013). Mechanisms Underlying the Attachment and Spreading of Human Osteoblasts: From Transient Interactions to Focal Adhesions on Vitronectin-Grafted Bioactive Surfaces. Acta biomater. 9, 6105–6115. 10.1016/j.actbio.2012.12.018 23261922

[B13] Burroughs L. Amer M. H. Vassey M. Koch B. Figueredo G. P. Mukonoweshuro B. (2021). Discovery of Synergistic Material-Topography Combinations to Achieve Immunomodulatory Osteoinductive Biomaterials Using a Novel *In Vitro* Screening Method: The ChemoTopoChip. Biomaterials 271, 120740. 10.1016/j.biomaterials.2021.120740 33714019

[B14] Cai S. Wu C. Yang W. Liang W. Yu H. Liu L. (2020). Recent Advance in Surface Modification for Regulating Cell Adhesion and Behaviors. Nanotechnol. Rev. 9, 971–989. 10.1515/ntrev-2020-0076

[B15] Chen E. Liu G. Zhou X. Zhang W. Wang C. Hu D. (2018). Concentration‐dependent, Dual Roles of IL‐10 in the Osteogenesis of Human BMSCsviaP38/MAPK and NF‐κB Signaling Pathways. FASEB J. 32, 4917–4929. 10.1096/fj.201701256rrr 29630408

[B16] Cheng K. Weng W. Wang H. Zhang S. (2005). *In Vitro* behavior of Osteoblast-like Cells on Fluoridated Hydroxyapatite Coatings. Biomaterials 26, 6288–6295. 10.1016/j.biomaterials.2005.03.041 15913766

[B17] Davies J. E. (2019). Is Osseointegration a Foreign Body Reaction? Hanover Park, Illinois, United States: Quintessence Publishing Co, Inc 4350 Chandler Drive.

[B18] Deng X. Hasan A. Elsharkawy S. Tejeda-Montes E. Tarakina N. V. Greco G. (2021). Topographically Guided Hierarchical Mineralization. Mater. Today Bio 11, 100119. 10.1016/j.mtbio.2021.100119 PMC8273417 34286238

[B19] Derby B. (2012). Printing and Prototyping of Tissues and Scaffolds. science 338, 921–926. 10.1126/science.1226340 23161993

[B20] Dresner-Pollak R. Gelb N. Rachmilewitz D. Karmeli F. Weinreb M. (2004). Interleukin 10-deficient Mice Develop Osteopenia, Decreased Bone Formation, and Mechanical Fragility of Long Bones. Gastroenterology 127, 792–801. 10.1053/j.gastro.2004.06.013 15362035

[B21] El Khassawna T. Serra A. Bucher C. H. Petersen A. Schlundt C. Könnecke I. (2017). T Lymphocytes Influence the Mineralization Process of Bone. Front. Immunol. 8, 562. 10.3389/fimmu.2017.00562 28596766 PMC5442173

[B22] Elsharkawy S. Al-Jawad M. Pantano M. F. Tejeda-Montes E. Mehta K. Jamal H. (2018). Protein Disorder-Order Interplay to Guide the Growth of Hierarchical Mineralized Structures. Nat. Commun. 9, 2145. 10.1038/s41467-018-04319-0 29858566 PMC5984621

[B23] Farré-Guasch E. Wolff J. Helder M. N. Schulten E. A. Forouzanfar T. Klein-Nulend J. (2015). Application of Additive Manufacturing in Oral and Maxillofacial Surgery. J. Oral Maxillofac. Surg. 73, 2408–2418. 10.1016/j.joms.2015.04.019 25966454

[B24] Fu X. Liu G. Halim A. Ju Y. Luo Q. Song G. (2019). Mesenchymal Stem Cell Migration and Tissue Repair. Cells 8, 784. 10.3390/cells8080784 PMC6721499 31357692

[B25] Guillaume O. Schmid T. Kluge K. Weber F. E. Richards R. G. Eberli U. (2019). Introduction of the Anspach Drill as a Novel Surgical Driller for Creating Calvarial Defects in Animal Models. J. Orthop. Res. 37, 1183–1191. 10.1002/jor.24265 30835898

[B26] Hasan A. Pandey L. M. (2020). Surface Modification of Ti6Al4V by Forming Hybrid Self-Assembled Monolayers and its Effect on Collagen-I Adsorption, Osteoblast Adhesion and Integrin Expression. Appl. Surf. Sci. 505, 144611. 10.1016/j.apsusc.2019.144611

[B27] Hasan A. Waibhaw G. Pandey L. M. (2018). Conformational and Organizational Insights into Serum Proteins during Competitive Adsorption on Self-Assembled Monolayers. Langmuir 34, 8178–8194. 10.1021/acs.langmuir.8b01110 29936836

[B28] Hay D. I. Moreno E. C. (2021). “Statherin and the Acidic Proline-Rich Proteins,” in Human Saliva: Clinical Chemistry and Microbiology. Editor Tenovuo J. O. (Boca Raton, FL: CRC Press), 131–150. 10.1201/9781003210399-5

[B29] Holgersen K. Dobie R. Farquharson C. vanʼt Hof R. Ahmed S. F. Hansen A. K. (2015). Piroxicam Treatment Augments Bone Abnormalities in Interleukin-10 Knockout Mice. Inflamm. bowel Dis. 21, 257–266. 10.1097/mib.0000000000000269 25569742

[B30] Jackson R. J. Patrick P. S. Page K. Powell M. J. Lythgoe M. F. Miodownik M. A. (2018). Chemically Treated 3D Printed Polymer Scaffolds for Biomineral Formation. ACS omega 3, 4342–4351. 10.1021/acsomega.8b00219 29732454 PMC5928486

[B31] Jeong J. Kim J. H. Shim J. H. Hwang N. S. Heo C. Y. (2019). Bioactive Calcium Phosphate Materials and Applications in Bone Regeneration. Biomater. Res. 23, 4–11. 10.1186/s40824-018-0149-3 30675377 PMC6332599

[B32] Jiang J. Liu W. Xiong Z. Hu Y. Xiao J. (2022). Effects of Biomimetic Hydroxyapatite Coatings on Osteoimmunomodulation. Biomater. Adv. 134, 112640. 10.1016/j.msec.2021.112640 35577692

[B33] Jung Y.-K. Kim G.-W. Park H.-R. Lee E.-J. Choi J.-Y. Beier F. (2013). Role of Interleukin-10 in Endochondral Bone Formation in Mice: Anabolic Effect via the Bone Morphogenetic Protein/Smad Pathway. Arthritis & Rheumatism 65, 3153–3164. 10.1002/art.38181 24022823

[B34] Karageorgiou V. Kaplan D. (2005). Porosity of 3D Biomaterial Scaffolds and Osteogenesis. Biomaterials 26, 5474–5491. 10.1016/j.biomaterials.2005.02.002 15860204

[B35] Khosravi N. Maeda A. Dacosta R. S. Davies J. E. (2018). Nanosurfaces Modulate the Mechanism of Peri-Implant Endosseous Healing by Regulating Neovascular Morphogenesis. Commun. Biol. 1, 72–13. 10.1038/s42003-018-0074-y 30271953 PMC6123776

[B36] Lebre F. Sridharan R. Sawkins M. J. Kelly D. J. O'Brien F. J. Lavelle E. C. (2017). The Shape and Size of Hydroxyapatite Particles Dictate Inflammatory Responses Following Implantation. Sci. Rep. 7, 2922. 10.1038/s41598-017-03086-0 28592868 PMC5462791

[B37] Lee E.-H. Kim J.-Y. Kweon H. Y. Jo Y.-Y. Min S.-K. Park Y.-W. (2010). A Combination Graft of Low-Molecular-Weight Silk Fibroin with Choukroun Platelet-Rich Fibrin for Rabbit Calvarial Defect. Oral Surg. Oral Med. Oral Pathology, Oral Radiology, Endodontology 109, e33–e38. 10.1016/j.tripleo.2009.12.043 20149696

[B38] Lee S. J. Lee D. Yoon T. R. Kim H. K. Jo H. H. Park J. S. (2016). Surface Modification of 3D-Printed Porous Scaffolds via Mussel-Inspired Polydopamine and Effective Immobilization of rhBMP-2 to Promote Osteogenic Differentiation for Bone Tissue Engineering. Acta biomater. 40, 182–191. 10.1016/j.actbio.2016.02.006 26868173

[B39] Liu Y. Rath B. Tingart M. Eschweiler J. (2020). Role of Implants Surface Modification in Osseointegration: A Systematic Review. J. Biomed. Mater Res. 108, 470–484. 10.1002/jbm.a.36829 31664764

[B40] Malhotra A. Habibovic P. (2016). Calcium Phosphates and Angiogenesis: Implications and Advances for Bone Regeneration. Trends Biotechnol. 34, 983–992. 10.1016/j.tibtech.2016.07.005 27481474

[B41] Mehrabanian M. Nasr-Esfahani M. (2011). HA/nylon 6,6 Porous Scaffolds Fabricated by Salt-Leaching/solvent Casting Technique: Effect of Nano-Sized Filler Content on Scaffold Properties. Int. J. Nanomedicine 6, 1651–1659. 10.2147/IJN.S21203 21904455 PMC3160951

[B42] Mori T. Kiyono T. Imabayashi H. Takeda Y. Tsuchiya K. Miyoshi S. (2005). Combination of hTERT and Bmi-1 , E6, or E7 Induces Prolongation of the Life Span of Bone Marrow Stromal Cells from an Elderly Donor without Affecting Their Neurogenic Potential. Mol. Cell Biol. 25, 5183–5195. 10.1128/mcb.25.12.5183-5195.2005 15923633 PMC1140572

[B43] Noskovicova N. Schuster R. Van Putten S. Ezzo M. Koehler A. Boo S. (2021b). Suppression of the Fibrotic Encapsulation of Silicone Implants by Inhibiting the Mechanical Activation of Pro-fibrotic TGF-β. Nat. Biomed. Eng. 5, 1437–1456. 10.1038/s41551-021-00722-z 34031559

[B44] Noskovicova N. Hinz B. Pakshir P. (2021a). Implant Fibrosis and the Underappreciated Role of Myofibroblasts in the Foreign Body Reaction. Cells 10, 1794. 10.3390/cells10071794 34359963 PMC8304203

[B45] Orciani M. Fini M. Di Primio R. Mattioli-Belmonte M. (2017). Biofabrication and Bone Tissue Regeneration: Cell Source, Approaches, and Challenges. Front. Bioeng. Biotechnol. 5, 17. 10.3389/fbioe.2017.00017 28386538 PMC5362636

[B46] Owen R. Bahmaee H. Claeyssens F. Reilly G. C. (2020). Comparison of the Anabolic Effects of Reported Osteogenic Compounds on Human Mesenchymal Progenitor-Derived Osteoblasts. Bioengineering 7, 12. 10.3390/bioengineering7010012 PMC7148480 31972962

[B47] Parithimarkalaignan S. Padmanabhan T. V. (2013). Osseointegration: An Update. J. Indian Prosthodont Soc. 13, 2–6. 10.1007/s13191-013-0252-z 24431699 PMC3602536

[B48] Petrie T. A. Reyes C. D. Burns K. L. García A. J. (2009). Simple Application of Fibronectin-Mimetic Coating Enhances Osseointegration of Titanium Implants. J. Cell. Mol. Med. 13, 2602–2612. 10.1111/j.1582-4934.2008.00476.x 18752639 PMC2819599

[B49] Prasad K. Bazaka O. Chua M. Rochford M. Fedrick L. Spoor J. (2017). Metallic Biomaterials: Current Challenges and Opportunities. Materials 10, 884. 10.3390/ma10080884 PMC5578250 28773240

[B50] Qu H. Fu H. Han Z. Sun Y. (2019). Biomaterials for Bone Tissue Engineering Scaffolds: A Review. RSC Adv. 9, 26252–26262. 10.1039/c9ra05214c 35531040 PMC9070423

[B51] Ran Q. Yang W. Hu Y. Shen X. Yu Y. Xiang Y. (2018). Osteogenesis of 3D Printed Porous Ti6Al4V Implants with Different Pore Sizes. J. Mech. Behav. Biomed. Mater. 84, 1–11. 10.1016/j.jmbbm.2018.04.010 29709846

[B52] Salazar F. Hall L. Negm O. H. Awuah D. Tighe P. J. Shakib F. (2016). The Mannose Receptor Negatively Modulates the Toll-like Receptor 4-aryl Hydrocarbon Receptor-Indoleamine 2,3-dioxygenase axis in Dendritic Cells Affecting T Helper Cell Polarization. J. Allergy Clin. Immunol. 137, 1841–1851. e1842. 10.1016/j.jaci.2015.10.033 26703454

[B53] Schmidlin P. R. Nicholls F. Kruse A. Zwahlen R. A. Weber F. E. (2013). Evaluation of Moldable,in Situhardening Calcium Phosphate Bone Graft Substitutes. Clin. Oral Impl. Res. 24, 149–157. 10.1111/j.1600-0501.2011.02315.x 22092691

[B54] Seyednejad H. Gawlitta D. Dhert W. J. A. Van Nostrum C. F. Vermonden T. Hennink W. E. (2011). Preparation and Characterization of a Three-Dimensional Printed Scaffold Based on a Functionalized Polyester for Bone Tissue Engineering Applications. Acta biomater. 7, 1999–2006. 10.1016/j.actbio.2011.01.018 21241834

[B55] Shah F. A. Thomsen P. Palmquist A. (2019). Osseointegration and Current Interpretations of the Bone-Implant Interface. Acta biomater. 84, 1–15. 10.1016/j.actbio.2018.11.018 30445157

[B56] Song R. Murphy M. Li C. Ting K. Soo C. Zheng Z. (2018). Current Development of Biodegradable Polymeric Materials for Biomedical Applications. Dddt Vol. 12, 3117–3145. 10.2147/dddt.s165440 PMC6161720 30288019

[B57] Tejeda-Montes E. Klymov A. Nejadnik M. R. Alonso M. Rodriguez-Cabello J. C. Walboomers X. F. (2014a). Mineralization and Bone Regeneration Using a Bioactive Elastin-like Recombinamer Membrane. Biomaterials 35, 8339–8347. 10.1016/j.biomaterials.2014.05.095 24996755

[B58] Tejeda-Montes E. Smith K. H. Poch M. López-Bosque M. J. Martín L. Alonso M. (2012). Engineering Membrane Scaffolds with Both Physical and Biomolecular Signaling. Acta biomater. 8, 998–1009. 10.1016/j.actbio.2011.09.005 21945830

[B59] Tejeda-Montes E. Smith K. H. Rebollo E. Gómez R. Alonso M. Rodriguez-Cabello J. C. (2014b). Bioactive Membranes for Bone Regeneration Applications: Effect of Physical and Biomolecular Signals on Mesenchymal Stem Cell Behavior. Acta biomater. 10, 134–141. 10.1016/j.actbio.2013.09.001 24035887

[B60] Tofail S. A. M. Koumoulos E. P. Bandyopadhyay A. Bose S. O’Donoghue L. Charitidis C. (2018). Additive Manufacturing: Scientific and Technological Challenges, Market Uptake and Opportunities. Mater. today 21, 22–37. 10.1016/j.mattod.2017.07.001

[B61] Trindade R. Albrektsson T. Tengvall P. Wennerberg A. (2016). Foreign Body Reaction to Biomaterials: On Mechanisms for Buildup and Breakdown of Osseointegration. Clin. implant Dent. Relat. Res. 18, 192–203. 10.1111/cid.12274 25257971

[B62] Turnbull G. Clarke J. Picard F. Riches P. Jia L. Han F. (2018). 3D Bioactive Composite Scaffolds for Bone Tissue Engineering. Bioact. Mater. 3, 278–314. 10.1016/j.bioactmat.2017.10.001 29744467 PMC5935790

[B63] Vallés G. García-Rey E. Saldaña L. García-Cimbrelo E. Vilaboa N. (2021). Wear of Hip Prostheses Increases Serum IGFBP-1 Levels in Patients with Aseptic Loosening. Sci. Rep. 11, 576. 10.1038/s41598-020-79813-x 33436773 PMC7804331

[B64] Wang C. Huang W. Zhou Y. He L. He Z. Chen Z. (2020). 3D Printing of Bone Tissue Engineering Scaffolds. Bioact. Mater. 5, 82–91. 10.1016/j.bioactmat.2020.01.004 31956737 PMC6962643

[B65] Wang X. Shah F. A. Palmquist A. Grandfield K. (2017). 3D Characterization of Human Nano-Osseointegration by on-axis Electron Tomography without the Missing Wedge. ACS Biomater. Sci. Eng. 3, 49–55. 10.1021/acsbiomaterials.6b00519 33429681

[B66] Zhang Q. Chen B. Yan F. Guo J. Zhu X. Ma S. (2014). Interleukin-10 Inhibits Bone Resorption: A Potential Therapeutic Strategy in Periodontitis and Other Bone Loss Diseases. BioMed Res. Int. 2014, 1–5. 10.1155/2014/284836 PMC3947664 24696846

[B67] Zhang X. Lou Q. Wang L. Min S. Zhao M. Quan C. (2019). Immobilization of BMP-2-Derived Peptides on 3D-Printed Porous Scaffolds for Enhanced Osteogenesis. Biomed. Mat. 15, 015002. 10.1088/1748-605x/ab4c78 31597124

[B68] Zhao X. Lui Y. S. Choo C. K. C. Sow W. T. Huang C. L. Ng K. W. (2015). Calcium Phosphate Coated Keratin-PCL Scaffolds for Potential Bone Tissue Regeneration. Mater. Sci. Eng. C 49, 746–753. 10.1016/j.msec.2015.01.084 25687004

[B69] Zhu G. Wang G. Li J. J. (2021). Advances in Implant Surface Modifications to Improve Osseointegration. Mater. Adv. 2 (21), 6901–6927. 10.1039/d1ma00675d

[B70] Zysset P. K. Guo X. E. Hoffler C. E. Moore K. E. Goldstein S. A. (1999). Elastic Modulus and Hardness of Cortical and Trabecular Bone Lamellae Measured by Nanoindentation in the Human Femur. J. biomechanics 32, 1005–1012. 10.1016/s0021-9290(99)00111-6 10476838

