# Supplementary material for: Mineralizing Coating on 3D Printed Scaffolds for the Promotion of Osseointegration
Source: Front Bioeng Biotechnol. 2022 Jun 27;10:836386. doi: 10.3389/fbioe.2022.836386 (PMC9271852; doi:10.3389/fbioe.2022.836386)
Supplement: Supplementary file 1 [file DataSheet1.docx]

**Mineralizing coating on 3D printed scaffolds for enhanced osseo-integration**

Abshar Hasan^1,2,3^, Romain Bagnol^4^, Robert Owen^1,2^, Arsalan Latif^5^, Hassan M. Rostam^5^, Sherif Elsharkawy^6^, Felicity R.A.J. Rose^1,2^, José Carlos Rodriguez-Cabello^7^, Amir M. Ghaemmaghami^5^, David Eglin^4,8,*^, Alvaro Mata^1,2,3,*^

^1^School of Pharmacy, University of Nottingham, NG7 2RD Nottingham, UK.

^2^Biodiscovery Institute, University of Nottingham, NG7 2RD Nottingham, UK.

^3^Department of Chemical and Environmental Engineering, University of Nottingham, NG7 2RD Nottingham, UK.

^4^Regenerative Orthopaedics, AO Research Institute, Clavadelerstrasse 8, 7270 Davos, Switzerland.

^5^ Immunology & Immuno-bioengineering Group, School of Life Sciences, University of Nottingham, NG7 2RD Nottingham, UK.

^6^Faculty of Dentistry, Oral & Craniofacial Sciences, King's College London, London SE1 9RT, UK.

^7^BIOFORGE Group, University of Valladolid, CIBER-BBN, Valladolid 47011, Spain.

^8^Ecole des Mines Saint-Etienne, F-42000 Saint-Étienne, France.

* Corresponding authors: [a.mata@nottingham.ac.uk](mailto:a.mata@nottingham.ac.uk) and [david.eglin@emse.fr](mailto:david.eglin@emse.fr)


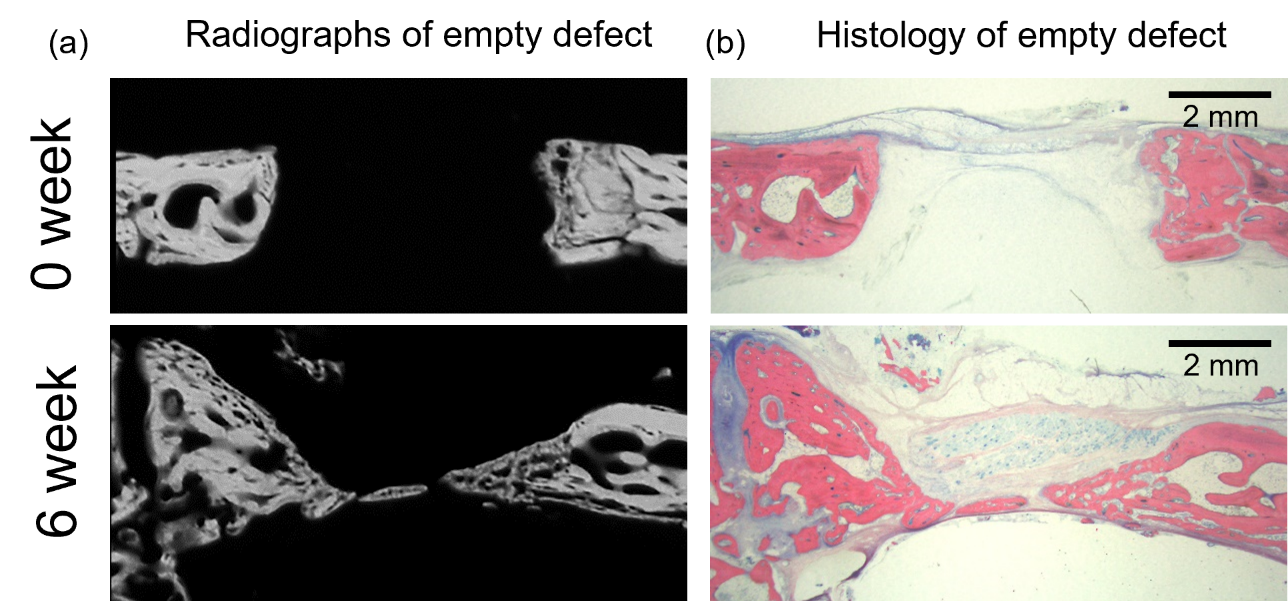


Figure S1. (a) Micro-CT and (b) histology images of new bone formation within the empty defect after 0 and 6 weeks.


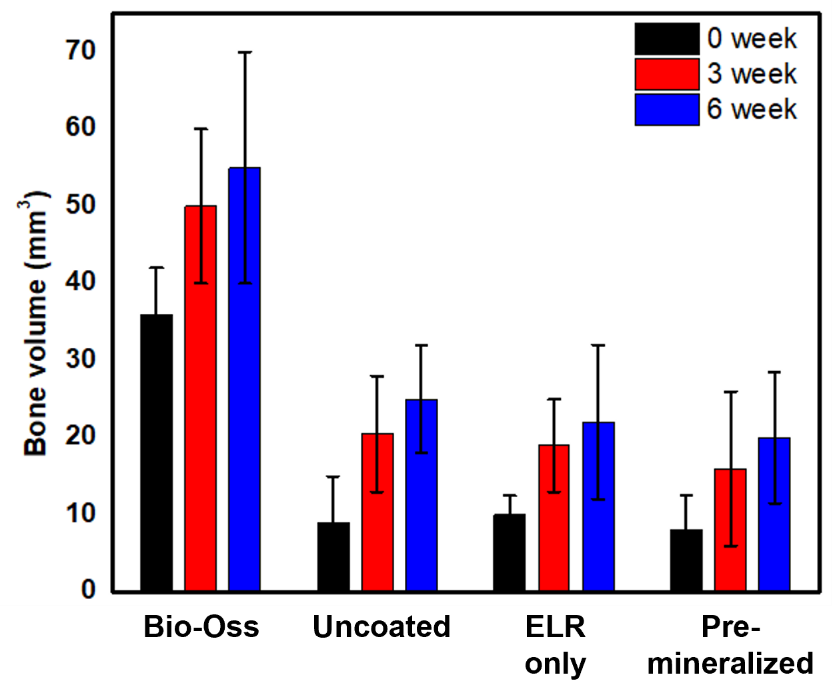


Figure S2. Bone volume of newly formed bone with positive control (Bio-Oss) and different test samples after 0, 3, and 6 weeks of implantation.
